# The Synergistic Effect of Nicotine and *Staphylococcus aureus* on Peri-Implant Infections

**DOI:** 10.3389/fbioe.2021.658380

**Published:** 2021-09-13

**Authors:** Yao Hu, Wen Zhou, Chengguang Zhu, Yujie Zhou, Qiang Guo, Xiaoyu Huang, Bina Yang, Biao Ren, Lei Cheng

**Affiliations:** ^1^ State Key Laboratory of Oral Diseases and West China School of Stomatology and National Clinical Research Center for Oral Diseases, Sichuan University, Chengdu, China; ^2^ Fujian Key Laboratory of Oral Diseases and Fujian Provincial Engineering Research Center of Oral Biomaterial and Stomatological Key Laboratory of Fujian College and University, School of Stomatology, Fujian Medical University, Fuzhou, China

**Keywords:** peri-implant infection, *Staphylococcus aureus*, nicotine, synergistic effect, RANKL

## Abstract

Smoking is considered a key risk factor for implant survival; however, how it interacts with the pathogens in peri-implant infections is not clear. Here, we identified that nicotine, the key component of cigarette smoking, can interact with *Staphylococcus aureus* and synergistically induce peri-implant infections in a rat osteolysis model. The nicotine–*S. aureus* combination group increased the gross bone pathology, osteolysis, periosteal reactions, and bone resorption compared to the nicotine or *S. aureus* single treated group (*p* < 0.05). Nicotine did not promote the proliferation of *S. aureus* both *in vitro* and *in vivo*, but it can significantly upregulate the expression of staphylococcal protein A (SpA), a key virulence factor of *S. aureus*. The nicotine–*S. aureus* combination also synergistically activated the expression of RANKL (receptor activator of nuclear factor-kappa B ligand, *p* < 0.05) to promote the development of peri-implant infections. The synergistic effects between nicotine and *S. aureus* infection can be a new target to reduce the peri-implant infections.

## Introduction

The dental implant, as an osseointegration technique, has become one of the most common strategies for prosthetic rehabilitation (Amornvit P, 2014). The osseointegration technique has also been extended to orthopedics, limb amputees, total joint replacements, maxillofacial reconstruction, and orbital prostheses due to its superiority in mobility and biocompatibility (Hebert et al., 2017; Depypere et al., 2020; Lu et al., 2020). However, complications have become a big challenge for their clinical practice. Peri-implant infections, as one of the major inflammatory complications, can cause suppuration, revision surgery, and even removal of the prostheses (Albrektsson T, 1994; Annibali S, 2008; Lindhe et al., 2008; Lang et al., 2011; Romano et al., 2013; Zimmerli and Sendi, 2017; Schwarz et al., 2018; Carrasco-Garcia et al., 2019; Kordbacheh Changi et al., 2019; Rokaya et al., 2020). The dental peri-implant infection prevalence was about 22%, while the global implant infection risk in orthopedic surgeries was about 2–5% (Derks and Tomasi, 2015; Berglundh et al., 2018; Dreyer et al., 2018; Ahn et al., 2019; Weinstein et al., 2020). Implant infections may increase by 20 times in high-risk patients, such as populations with diabetes and immunosuppressed diseases. Peri-implant infections dramatically increased the medical costs and human sufferings (Seebach and Kubatzky, 2019; Depypere et al., 2020; Zhou et al., 2020).

*Staphylococcus aureus* is commonly recognized as the key pathogenic agent for the early failure of implants due to its ability to attach onto different titanium surfaces (Harris and Richards, 2004; Cortizo et al., 2012; Shrestha et al., 2012; Persson and Renvert, 2014; Zhao et al., 2014; Thomas; Thurnheer and Belibasakis, 2016; Oliveira et al., 2018; Sahrmann et al., 2020). Moreover, *S. aureus* can also invade and persist in human osteoblasts to cause cell death and osteolysis (Bost et al., 1999; Alexander and Hudson, 2001). The staphylococcal protein A (SpA) is an important virulence factor of *S. aureus*. SpA can be recognized by the host immune system to induce the differentiation of osteoclast and can interact with the tumor necrosis factor receptor superfamily, member 1A (TNFR-1) on the surface of the osteoblast to activate the expression of RANKL (receptor activator of nuclear factor-kappa B ligand), which then promotes osteoclastogenesis (Somayaji et al., 2008; Claro et al., 2011; Jin et al., 2013; Kamohara et al., 2020).

Smoking is another risk factor for various diseases including dental peri-implant diseases and bone fragility (Kasat and Ladda, 2012; Barao et al., 2015; Carvalho and Rossi, 2019; Kordbacheh Changi et al., 2019; Vignoletti et al., 2019; Anderson et al., 2020). Bain CA (1993) reported an implant failure rate of 11.28% in smokers but only 4.76% in nonsmokers. The peri-implant disease symptoms were all worse in cigarette smokers than that in nonsmokers (ALHarthi et al., 2018). Nicotine, among more than 4,000 harmful substances in cigarette, is one of the key components (Barao et al., 2015). Nicotine regulated bone formation through the RANK-RANKL-OPG (osteoprotegerin) system (Lappin et al., 2007; Tang et al., 2009). Rats with cigarette smoking exhibited higher RANKL/OPG ratio to promote osteoclast formation (Giorgetti et al., 2010). Nicotine could also induce the osteoclast differentiation and enhance the resorbing ability of osteoclasts through RANKL (Costa-Rodrigues et al., 2018). However, the interactions between nicotine and *S. aureus* infection are not clear. Therefore, we investigated the interaction between *S. aureus* and nicotine on the initiation of peri-implant infections and bone loss in a murine osteolysis model in this study, and their effects on osteoblasts were also evaluated.

## Materials and Methods

### Bacteria Strains and Cultivation

*S. aureus* ATCC 25923 was used in this study (Inoue et al., 2017; Ao et al., 2019). Strains were maintained on TSB plates (3% trypticase soy broth, 2% agar). Single colonies were subjected to a liquid TSB medium incubating at 37°C with 150 rpm agitation overnight. *S. aureus* cells were collected by centrifugation at 5,000 r/min, 4°C for 10 min. Then, the final *S. aureus* suspension was adjusted to the desired concentration in the TSB medium or in PBS. For the nicotine–*S. aureus* coculture experiment, optical densities of *S. aureus*–nicotine (nicotine concentration: 1 μM) (Costa-Rodrigues et al., 2018) or *S. aureus* alone were detected by using a spectrophotometer every half-hour, and growth curves were drawn. The *S. aureus*–nicotine combination and the single *S. aureus* overnight cultures were used for real-time quantitative polymerase chain reaction (RT-qPCR).

### The Murine Model

All animal works were conducted in strict accordance with the guidelines of the Ethics Committee of West China School of Sichuan University, and the protocols were fully approved by this Agency (license number WCHSIRB-D-2020-415).

Ti rods of 1.5 mm diameter and 20 mm height were used in this study. Forty Sprague Dawley (female, 12 weeks old) rats were firstly divided into two groups: Ti rods with PBS and Ti rods inoculated with *S. aureus* (concentration: 10^8^ CFU/ml) (Zhou et al., 2020). Ti rods with PBS and Ti rods with *S. aureus* were implanted into the left femurs of the rats as previously described (Diefenbeck et al., 2016; Nie et al., 2017). Then, ten randomly assigned rats from each group were subcutaneously injected with nicotine (2 mg/kg) once a day (Milovanovic et al., 2018; Ullrich et al., 2020), and the other ten were injected with PBS as control. There were four groups in total: PBS as the control group, *S. aureus* group, nicotine group, and *S. aureus*-nicotine combination group. X-ray analysis was performed on each rat, and radiographic scores were calculated one day or three weeks after surgery (*n* = 5) (Zhou et al., 2020). The rats were sacrificed three weeks after surgery, and the femurs were collected for gross pathology scoring and colony-forming unit (CFU) counts of bacteria dwelling in Ti rods as well as in bone tissues (*n* = 5) (Yang et al., 2016). Five femurs of each group were scanned with microcomputed tomography (micro-CT).

### Microcomputed Tomography

Rats were sacrificed three weeks after implantation, and the femurs were collected, fixed in 4% buffered formaldehyde, and scanned with a high-resolution micro-CT (SCANCO Medical AG, μCT 50, Brüttisellen, Switzerland) at a voxel size of 20 μm. The shafts of the femurs were chosen as the “region of interest” (ROI). Three-dimensional high-resolution (3D) images were obtained. Mean trabecular thickness (Tb.Th) and bone volume/total volume (BV/TV) were analyzed.

### Cell Lines and Cultivation

MC3T3-E1 preosteoblastic cell line was obtained from the State Key Laboratory of Oral Diseases. Cells were cultured in Minimum Essential Medium α (α-MEM, Gibco) supplemented with 10% fetal bovine serum (FBS, Gibco) and 1% penicillin–streptomycin at 37°C in the presence of 5% CO_2_. For the coculture experiment, *S. aureus* or the nicotine-treated *S. aureus* were treated as described previously (Kavanagh et al., 2018). For the nicotine and *S. aureus*–nicotine combination group, the culture medium was supplemented with 1 μM nicotine. Cells were harvested for RT-qPCR (Widaa et al., 2012; Zheng et al., 2019).

### Relative Quantification of Differentially Expressed Genes by RT-qPCR

The relative expressions of *spa* and RANKL were examined. Total RNA from *S. aureus* treated with or without nicotine (concentration: 1 μM) (Costa-Rodrigues et al., 2018) and MC3T3-E1 cells treated with or without nicotine and/or *S. aureus* was extracted with 1 ml TRIZol reagent (Invitrogen, United States) followed by the manufacturer’s instructions. cDNA was synthesized according to the One Step RNA PCR kit (Takara Inc.) protocols. The RT-qPCR were then proceeded following the SYBR® PremixEx TaqTM kit (Takara Inc.) two-step strategy: 1) 95°C for 30 s; 2) 40 PCR cycles (95°C for 5 s, a gene-specific annealing temperature for 30 s). All primer sequences used are listed in Table 1(Zheng et al., 2019), and 16S rDNA and β-actin were chosen as the reference genes. RT-qPCRs were run on LightCycler 480 II (Roche, Basel, Switzerland). The gene expression level relative to the calibrator was expressed as 2^−ΔΔCT^.

**TABLE 1:** Specific primers used for RT-qPCR.

Primers	Sequences
16s rDNA	Forward: 5’-GTA​GGT​GGC​AAG​CGT​TAT-3’
	Reverse: 5’-GGT​GTT​CCT​CCA​TAT​CTC​TG-3’
*spa*	Forward: 5’-ACA​ACA​ACA​AGC​CTG​GTA​A-3’
	Reverse: 5’-AGT​AGT​GCC​GTT​TGC​TTT-3’
β-Actin	Forward: 5’-GTG​CTA​TGT​TGC​TCT​AGA​CTT​CG-3’
	Reverse: 5’-ATG​CCA​CAG​GAT​TCC​ATA​CC-3’
RANKL	Forward: 5’-GGA​AGC​GTA​CCT​ACA​GAC​TAT​C-3’
	Reverse: 5’-AAA​GTG​GAA​TTC​AGA​ATT​GCC​C-3’

## Results

### Nicotine and *S. aureus* Synergistically Increased the Gross Bone Pathology

**FIGURE 1:**
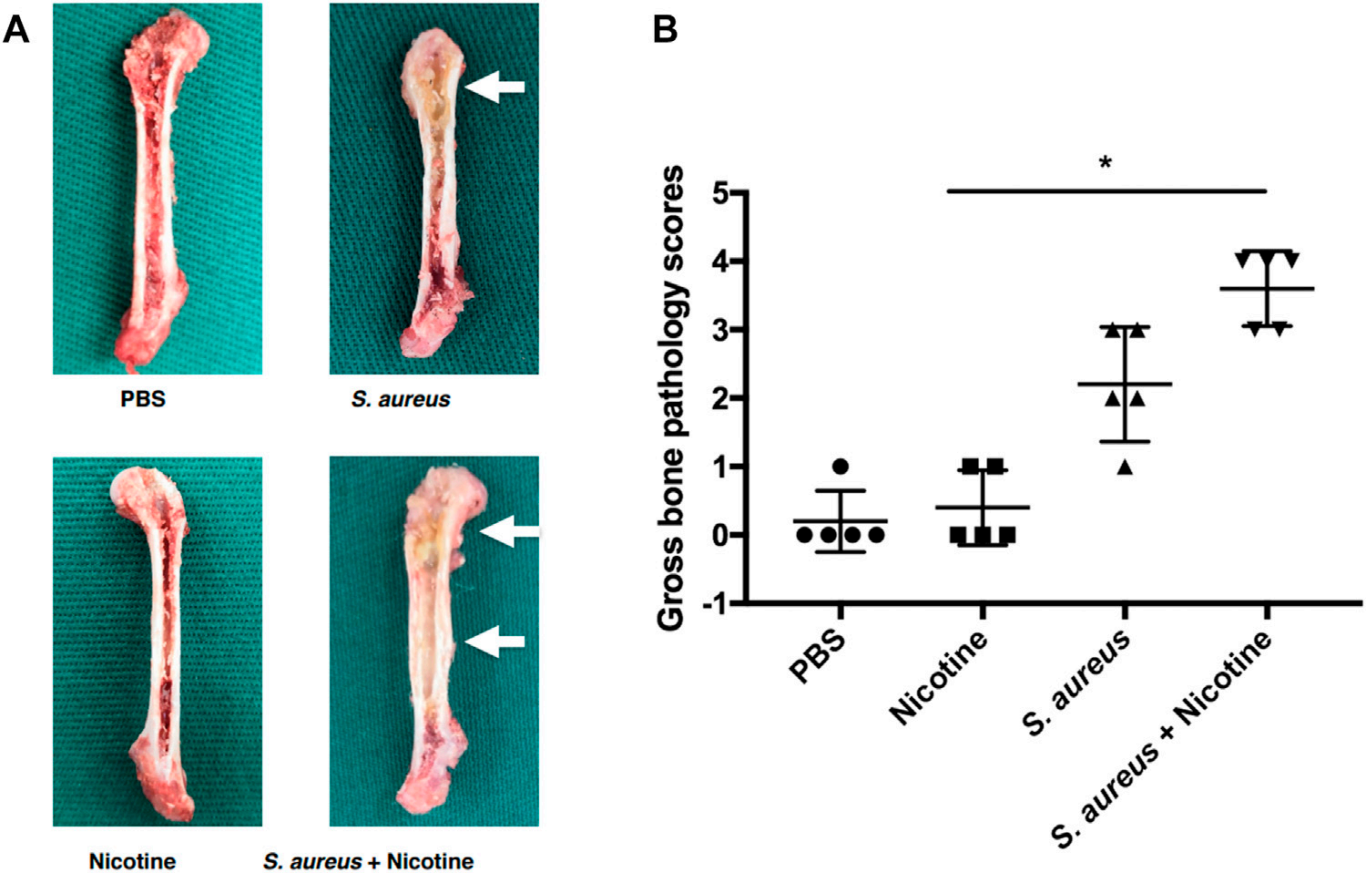
Nicotine and *S. aureus* synergistically increased the gross bone pathology. **(A)** Gross appearance of the rat femurs three weeks after implantation. Abscess and widening of the shaft are pointed out with white arrows. **(B)** Gross bone pathology scores of rat femurs. *p < 0.05.

A gross bone pathology assessment was performed to evaluate the clinical symptoms. Clinical symptoms of pyogenic infections such as abscess and shaft widening could be seen in *S. aureus* and its combination with nicotine groups after three weeks as pointed out by white arrows (Figure 1A), while the diaphysis was infected only in the nicotine–*S. aureus* combination group (Figure 1A). Gross bone pathology scores indicated that the average scores from both the *S. aureus* and nicotine–*S. aureus* combination groups significantly increased and the nicotine–*S. aureus* combination group caused the severest symptoms (Figure 1B). However, there was no significant difference between the nicotine and control groups (Figure 1B).

### Nicotine and *S. aureus* Synergistically Induced the Osteolysis and Periosteal Reactions

**FIGURE 2:**
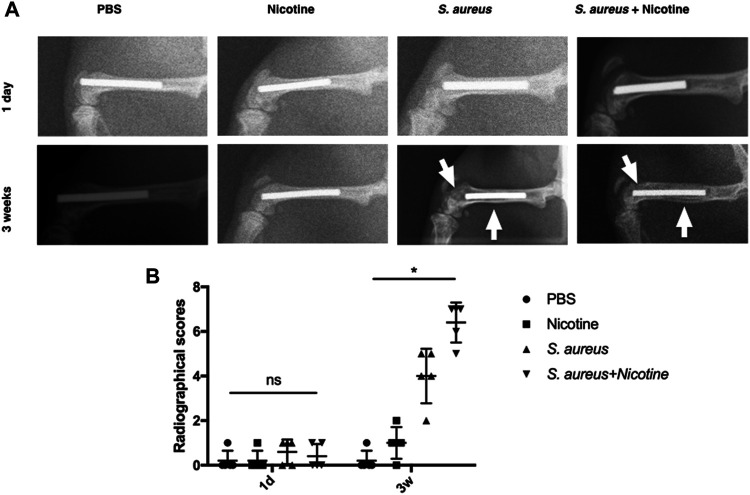
Nicotine and *S. aureus* synergistically induced the osteolysis and periosteal reactions. **(A)** X-ray images of rat femurs with Ti rod implants at one day or three weeks after surgery. White arrows indicate osteolytic lesions and cortical bone destruction. **(B)** Radiographical scores of X-ray images. *p < 0.05. ns: *p* ≧ 0.05.

To further determine the synergistic effect of nicotine and *S. aureus*, X-ray analysis was performed for the radiographical evaluation at day one and week three. The *S. aureus* group and the nicotine–*S. aureus* combination group both exhibited obvious symptoms of osteolysis and periosteal reactions after three weeks as indicated by white arrows (Figure 2A). There was a large scale of bone resorption and sequestrum formation in the nicotine–*S. aureus* combination group, while little bone infection was observed in the nicotine group (Figure 2A) indicating the synergism between nicotine and *S. aureus*. There was no significant difference in the radiographical scores among the groups at one day after surgery, but the radiographical scores of the *S. aureus* and the nicotine–*S. aureus* combination groups were significantly higher after three weeks (Figure 2B).

### Nicotine and *S. aureus* Synergistically Promoted the Bone Resorption

**FIGURE 3:**
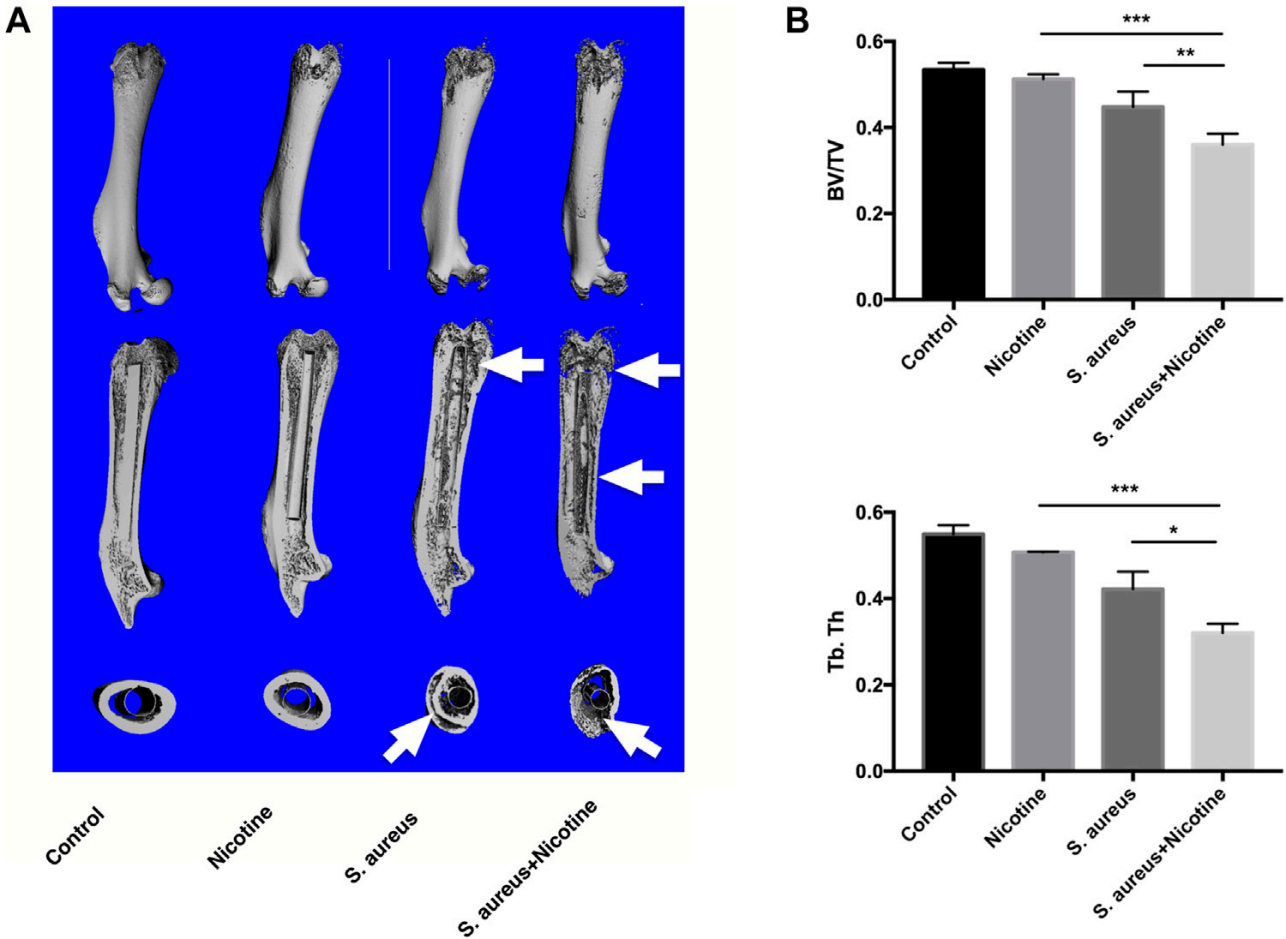
Nicotine and *S. aureus* synergistically promoted the bone resorption. **(A)** 3D micro-CT images of rat femurs at three weeks after surgery. Osteolytic lesions and cortical bone destruction are pointed out by white arrows. **(B)** BV/TV and Tb.Th of the selected regions evaluated by micro-CT. *p < 0.05. **p < 0.01. ***p < 0.001.

High-resolution micro-CT assessment was conducted to qualitatively and quantitatively analyze the bone tissues of rat femurs three weeks after implantation. 3D images indicated strong osteolysis and cortical bone absorption in the *S. aureus* and nicotine–*S. aureus* combination groups, while few bone infection signs were observed in the nicotine group (Figure 3A). The *S. aureus* group also showed reduced BV/TV and Tb.Th scores, but there was no significant difference between the nicotine and control groups (Figure 3B). The BV/TV and Tb.Th significantly decreased in the nicotine–*S. aureus* combination groups compared with the other three groups (Figure 3B) indicating their synergism on the bone resorption.

### Nicotine Showed No Impact on the Proliferation of *S. aureus*

**FIGURE 4:**
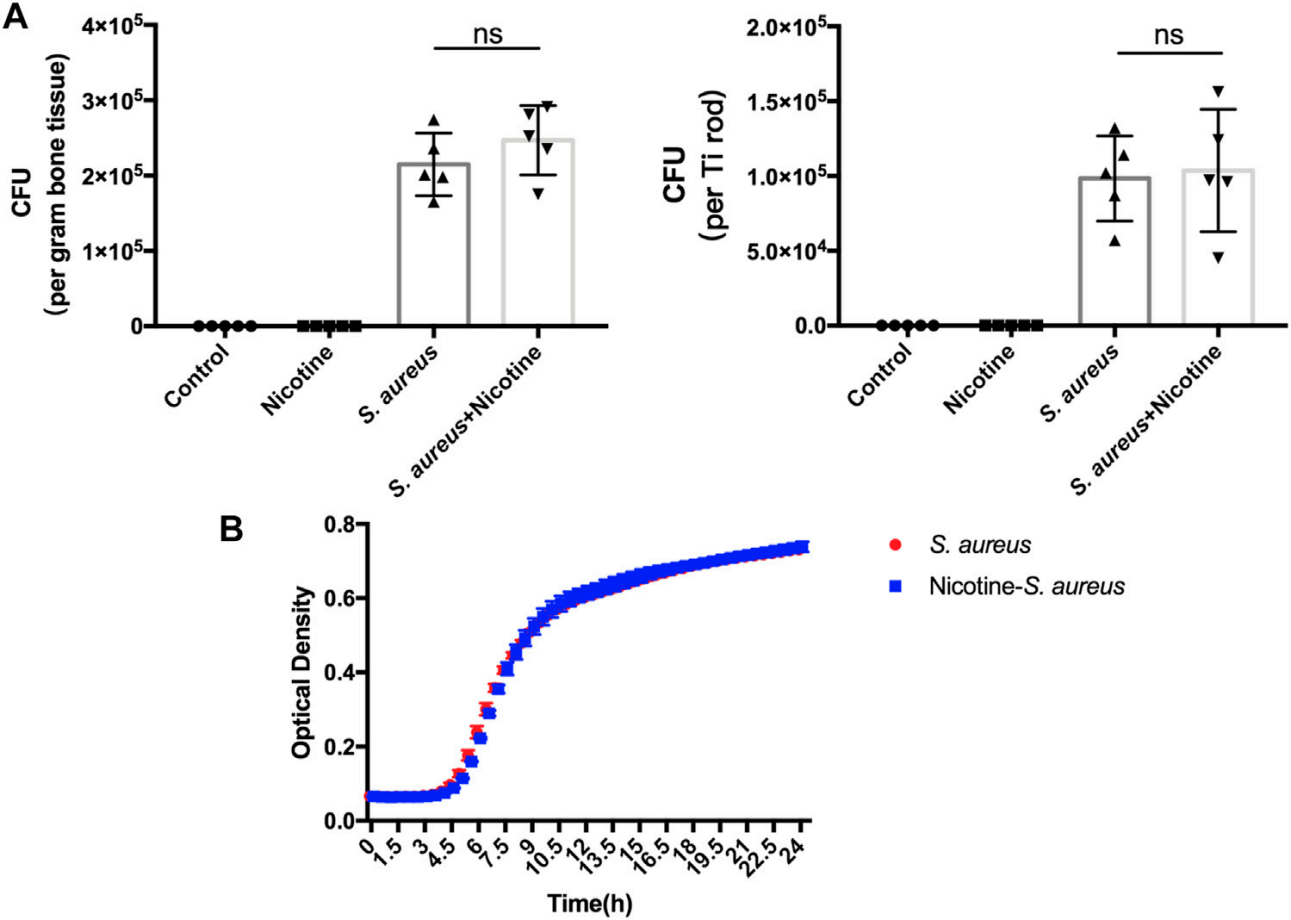
Nicotine showed no impact on the proliferation of *S. aureus*. **(A)** Quantitative analysis of *S. aureus* dwelling in Ti rods or in the bone tissue. **(B)** Growth curves of *S. aureus* and nicotine–*S. aureus*. ns: *p* ≧ 0.05.

We further evaluated whether the synergistic effect is achieved through nicotine’s impact on the proliferation of *S. aureus*. The CFU counts of bacteria dwelling in the bone tissues and Ti rods were calculated. There was no significant difference in the bacteria load between the *S. aureus* and nicotine–*S. aureus* combination groups (Figure 4A) indicating that nicotine at this concentration did not affect the proliferation of *S. aureus*. To verify if nicotine could influence the proliferation of *S. aureus in vitro*, we detected the optical density of the *S. aureus* and nicotine–*S. aureus* combination groups every half-hour and the growth curves were drawn. As shown in Figure 4B, no significant difference was observed between these two groups.

### Nicotine Upregulated the Virulence of *S. aureus* and Synergistically Activated the RANKL Pathway

**FIGURE 5:**
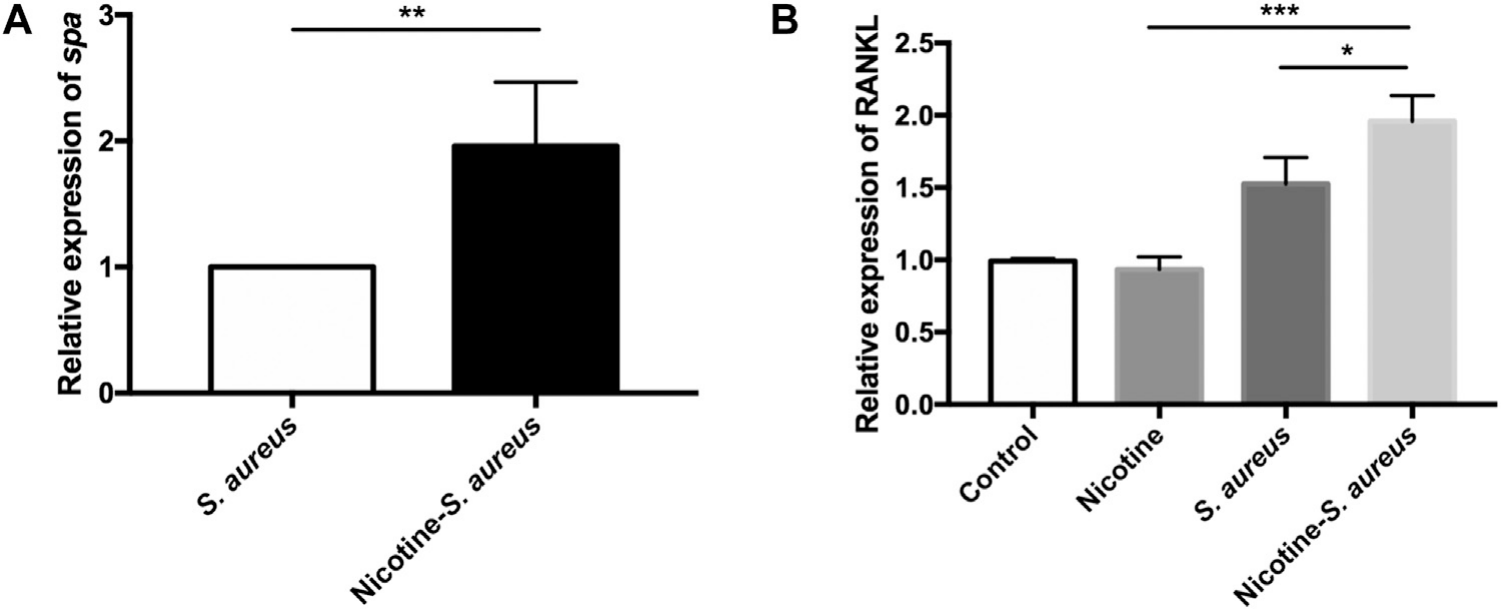
Nicotine upregulated the virulence of *S. aureus* and synergistically activated the RANKL pathway. **(A)** Relative expression of *spa* in *S. aureus* or nicotine-treated *S. aureus* measured by RT-qPCR. All gene expression levels were normalized by 16s rRNA gene expression. **(B)** Relative expression of nuclear factor-kappa B ligand (RANKL) in MC3T3-E1 in the presence of nicotine, *S. aureus*, or both, measured by RT-qPCR. All gene expression levels were normalized by β-actin gene expression. *p < 0.05. **p < 0.01. ***p < 0.001.

To explore the synergistic mechanisms, we examined the relative expression of *spa* from the nicotine–*S. aureus* combination and *S. aureus* groups. Interestingly, the relative expression of *spa* was significantly upregulated in the nicotine–*S. aureus* combination group compared with *S. aureus* alone (Figure 5A). We then tested if nicotine and *S. aureus* could synergistically promote RANKL expression. *S. aureus* alone increased the RANKL expression, but there was no significant difference between the nicotine and control groups (Figure 5B). The relative expression was significantly upregulated in the nicotine–*S. aureus* combination group compared with the other three groups (Figure 5B) indicating their synergism on the activation of the RANKL pathway.

## Discussion

Peri-implant diseases have been recognized as a global health challenge in dental prosthetic rehabilitation, orthopedics, joint replacements, etc. The microbial biofilm accumulation has been considered as the essential initiator of peri-implant diseases (Lindhe et al., 2008; Oliveira et al., 2018), but the mechanisms for peri-implant infection progress are still not fully characterized because of the complex interactions between different risk factors.

Peri-implant infections are characterized by osteolysis and bone loss. Osteoclasts play an essential role in this process as they are the only bone-resorbing cells (Albrektsson T, 1994; Kadoya et al., 1997; Annibali S, 2008; Uehara et al., 2018; Rokaya et al., 2020). The bone resorption is proved to be decided by the activity and survival of osteoclasts (Goodman and Gallo, 2019). The RANK-RANKL-OPG pathway is critical for osteoclast maturation (Xing et al., 2012). Briefly, RANKL expressed by osteoblasts activated the RANK on the surface of osteoclast precursors and then promoted the activation of NF-κB (nuclear factor-kappa B) to regulate the osteoclast differentiation, activation, and survival (Altaf and Revell, 2013; Park et al., 2017). According to previous studies, nicotine and *S. aureus* could both participate in this pathway to some extent leading to osteoclast activation (Somayaji et al., 2008; Giorgetti et al., 2010; Claro et al., 2011; Jin et al., 2013; Costa-Rodrigues et al., 2018; Kamohara et al., 2020). In our study, we verified the interactions between nicotine and *S. aureus* infection through a murine model. Their synergistic effects on peri-implant infections and the activation of RANKL were observed (Figures 1–3, 5).

The impacts of nicotine on bone resorption are controversial. Most scholars believed that cigarette smoking had a negative influence on bone healing while nicotine alone did not have such effect (Stefani et al., 2002; Cesar-Neto et al., 2003; Giorgetti et al., 2010; Zhu et al., 2020). However, nicotine administrated alone was reported to induce the osteoclastogenesis in another study (Ullrich et al., 2020). In our study, nicotine administrated alone induced few osteolysis and no significant difference was observed between the nicotine and control groups. However, it synergized with *S. aureus* infections to promote the gross bone pathology, osteolysis, periosteal reactions, and bone resorption (Figures 1–3). Our results also showed that nicotine had no impact on the proliferation of *S. aureus* both *in vivo* and *in vitro* (Figure 4) suggesting that the synergistic effect was not a result of the bacteria burden. Costa-Rodrigues et al. (2018) found that nicotine could induce osteoclast differentiation and enhance the resorbing ability of osteoclasts through the RANKL pathway. The virulence factor SpA of *S. aureus* could also interact with TNFR-1 on the osteoblast surface to promote the expression of RANKL, so we evaluated whether nicotine could facilitate *spa* expression to activate the RANKL pathway. We found that nicotine significantly upregulated the expression of *spa* in *S. aureus* and then significantly activated the RANKL pathway (Figure 5). Here, we identified another way nicotine participated in promoting osteoclastogenesis and bone resorption. To further investigate the detailed mechanisms, the transcriptome, proteome, and metabolome will be studied in the future.

The effects of nicotine on host cells may be dose dependent. An *in vitro* study examined the direct effect of nicotine on RAW264.7 cells and bone marrow cells, and the results demonstrated that 10^-5^ M to 10^-3^ M nicotine reduced the bone resorption by suppressing V-ATPase d2, cathepsin K and MMP-9 expression, and actin reorganization (Tanaka et al., 2013). In our study, we used 1 μM nicotine to treat MC3T3-E1 cells as suggested previously (Costa-Rodrigues et al., 2018). This dosage of nicotine was representative of the concentrations observed in the plasma and saliva of smokers. We found that this dosage of nicotine was able to synergize with *S. aureus* to activate RANKL expressions. Another study employed 2 mg/ml of nicotine to treat *S. aureus* and found that the biofilm mass was promoted (Shi et al., 2019). However, the biofilm contained increased numbers of dead *S. aureus* cells and the agr-dependent virulence of *S. aureus* was significantly reduced (Shi et al., 2019). This concentration of nicotine was higher than that in serum and was cytocidal to host cells and *S. aureus* cells in our previous results. We believe that the nicotine level is important for the investigation of the correlations among nicotine, microbiota, and the host.

In addition to *S. aureus*, numerous studies have also found that Gram-negative pathogens could play some roles in peri-implant diseases, such as *Veillonella* sp. spirochetes, *Actinobacillus actinomycetemcomitans, Porphyromonas gingivalis,* and *Prevotella intermedia* (Lafaurie et al., 2017; Teles, 2017; Sahrmann et al., 2020; Kotsakis and Olmedo, 2021). It has been recognized that smokers are more susceptible to *P. gingivalis* than nonsmokers and that nicotine may impact *P. gingivalis*’s inflammatory effect (Baek et al., 2012; Cogo et al., 2012; Kashiwagi et al., 2012). The interactions between nicotine and other microbial species or multispecies are also important in the development of peri-implant diseases, and we will investigate that in the future.

In conclusion, our results indicated that nicotine and *S. aureus* can synergistically induce peri-implant infections. Nicotine upregulated the virulence gene *spa* in *S. aureus* to increase the RANKL expression in osteoblast precursors. Our results highlighted that targeting the interaction between nicotine and *S. aureus* was a practical way to reduce the peri-implant infections, especially in smokers.

## Data Availability

The original contributions presented in the study are included in the article/Supplementary Material; further inquiries can be directed to the corresponding authors.
